# Efficacy of early controlled motion of the ankle compared with no motion after non-operative treatment of an acute Achilles tendon rupture: study protocol for a randomized controlled trial

**DOI:** 10.1186/s13063-016-1697-2

**Published:** 2016-11-29

**Authors:** Kristoffer Weisskirchner Barfod, Maria Swennergren Hansen, Per Holmich, Anders Troelsen, Morten Tange Kristensen

**Affiliations:** 1 Department of Orthopedic Surgery, Clinical Orthopedic Research Hvidovre, Copenhagen University Hospital Hvidovre, Kettegård Allé 30, Hvidovre, 2650 Denmark; 2 Physical Medicine and Rehabilitation Research - Copenhagen (PMR-C), Department of Physiotherapy and Occupational Therapy, Copenhagen University Hospital Hvidovre, Kettegård Allé 30, Hvidovre, 2650 Denmark; 3 Sports Orthopedic Research Center - Copenhagen (SORC-C), Arthroscopic Center, Department of Orthopedic Surgery, Copenhagen University Hospital Hvidovre, Hvidovre, Denmark

**Keywords:** Achilles tendon, Achilles tendon rupture, Non-operative treatment, Dynamic mobilization, Early controlled motion

## Abstract

**Background:**

Early controlled ankle motion is widely used in the non-operative treatment of acute Achilles tendon rupture, though its safety and efficacy have never been investigated in a randomized setup. The objectives of this study are to investigate if early controlled motion of the ankle affects functional and patient-reported outcomes.

**Methods/design:**

The study is performed as a blinded, randomized, controlled trial with patients allocated in a 1:1 ratio to one of two parallel groups. Patients aged from 18 to 70 years are eligible for inclusion. The intervention group performs early controlled motion of the ankle in weeks 3–8 after rupture. The control group is immobilized. In total, 130 patients will be included from one big orthopedic center over a period of 2½ years. The primary outcome is the patient-reported Achilles tendon Total Rupture Score evaluated at 12 months post-injury. Secondary outcome measures are the heel-rise work test, Achilles tendon elongation, and the rate of re-rupture. The primary analysis will be conducted as intention-to-treat analyses.

**Discussion:**

This trial is the first to investigate the safety and efficacy of early controlled motion in the treatment of acute Achilles tendon rupture in a randomized setup. The study uses the patient-reported outcome measure, the Achilles tendon Total Rupture Score, as the primary endpoint, as it is believed to be the best surrogate measure for the tendon’s actual capability to function in everyday life.

**Trial registration:**

ClinicalTrials.gov: NCT02015364. Registered on 13 December 2013.

**Electronic supplementary material:**

The online version of this article (doi:10.1186/s13063-016-1697-2) contains supplementary material, which is available to authorized users.

## Background

One out of three patients has a poor outcome after treatment for acute Achilles tendon rupture [1, 2]. It is a frequent (20 to 32 per 100,000 per year) and potentially debilitating injury that typically affects young active adults [3, 4]. Consequently, there are great socioeconomic benefits in optimizing treatment and shortening recovery.

There is currently no consensus regarding the best treatment for acute Achilles tendon rupture. Traditionally, operative treatment has been considered superior; however, recent studies show that non-operative treatment is a safe treatment which leads to good results [5–7]. For this reason there has been a transition towards greater use of non-operative treatment in many orthopedic departments [8].

However, the optimal non-operative treatment protocol has yet to be clarified. Typically the treatment consists of 8 weeks of immobilization in a cast or orthosis with rehabilitation of the calf muscles and Achilles tendon beginning after week 8 [9, 10]. Since the 1980s the impact of early controlled motion of the ankle joint has been discussed [11–13]. In theory, early controlled motion of the tendon leads to a better and faster healing due to, inter alia, the release of growth factors [14]. Clinical studies looking at flexor tendons have found controlled early movement to promote faster and better healing [15, 16]. Animal studies have shown a three times increased strength in mobilized Achilles tendons compared to the immobilized ones [17]. Three randomized studies of patients with acute Achilles tendon rupture have compared operative and non-operative treatment using a dynamic rehabilitation protocol in both groups [18–20]. They show good results for both operative and non-operative treatment. In 2007 Twaddle and Poon concluded that: ”possibly, controlled early motion is the important factor in optimizing outcomes in patients with acute Achilles tendon rupture and surgery makes no difference to the outcome apart from increasing the risk of local infection” [18].

In 1992 Saleh et al. investigated the effect of early controlled ankle motion in non-operative treatment of acute Achilles tendon rupture in a semi-randomized study of 40 patients [11]. The group which was allowed early controlled motion had improved mobility of the ankle joint after 3 and 6 months but not after 12 months. There were no other significant differences between the groups. Since then, various randomized trials of varying quality have studied the effect of early controlled motion after operative treatment [12, 13, 21, 22]. They find increased mobility of the ankle joint within the first 6 months but otherwise no significant differences. One study finds a less pronounced elongation of the Achilles tendon in the group with early controlled motion [23].

Meanwhile, animal studies have shown that early controlled motion of the ankle joint can lead to elongation of the Achilles tendon [24], and elongation of the Achilles tendon has been shown to cause decreased push-off strength and thus a poor functional outcome [23, 25].

Several Nordic hospitals have, on the basis of the preceding results, changed their treatment algorithms to feature early controlled motion [8]. However, it has never been investigated, involving patients in a randomized setup, whether early controlled motion of the ankle joint is beneficial for the healing of the Achilles tendon in a non-operative treatment protocol.

With this project we wish to investigate the effect of early controlled motion of the ankle joint from day 14 following non-operative treatment. We will compare this to a traditional treatment protocol where the ankle joint is immobilized for 8 weeks. Full weight bearing is allowed from day 14 in both groups.

Hypothesis: The optimal loading of the Achilles tendon, and thereby the optimal healing condition, is achieved by early controlled motion of the ankle joint. This will result in a strong tendon with a shorter length than for immobilized ankle joints. The patient-allowed motion of the ankle will experience a better functional outcome and a better patient-reported health in comparison with immobilization.

Null hypothesis: There is no difference in the functional outcome or in patient-reported outcome when early controlled motion of the ankle joint is compared to immobilization.

## Methods/design

This is a report of the second version of the trial protocol dated 26 March 2014. Any important protocol modifications will be communicated through ClinicalTrials.gov, the Ethical Review Board, and the *Trials* journal. The protocol was developed in accordance with the guidelines and checklists for Standard Protocol Items: Recommendations for Interventional Trials (SPIRIT; see Additional file 1) and Consolidated Standards of Reporting Trials (CONSORT).

### Design

The study is performed as a blinded, randomized, controlled trial (RCT) with patients allocated in a 1:1 ratio to one of two parallel groups. In total 130 patients will be included.

### Objective

The primary objective of the study is to investigate if early controlled motion of the ankle in weeks 3–8 post-injury affects the patient-reported outcome after non-operative treatment of acute Achilles tendon rupture compared to patients not allowed any motion of the ankle.

### Primary outcome measure

The primary outcome measure is the Achilles tendon Total Rupture Score (ATRS) evaluated at 12 months post-injury. The ATRS is a patient-reported score developed to assess symptoms and physical activity after treatment for acute Achilles tendon rupture [26].

### Secondary outcome measures

The secondary outcome measures are listed below. (The timing of the assessments is shown in Fig. 3.)

- Heel-rise work test: Endurance test where the patient stands on one leg and lifts the heel up and down until exhaustion. The number and the height of the heel-rises are counted and measured. The results are then compared to the weight of the patient, and the total work is estimated. The MuscleLab ® (Ergo Test Technology, Oslo, Norway) measurement system is used [26].
- Single heel-rise test: The patient stands with the foot in 10 degrees of dorsiflexion. It is recorded whether the patient is able to make a single heel-rise on the injured leg. The heel-rise is acknowledged if the heel can be lifted at least 2 cm with stretched knee. The MuscleLab ® (Ergo Test Technology, Oslo, Norway) measurement system is used [27].
- Ultrasonographic tendon length: The length between the calcaneus and the distal tip of the medial head of the gastrocnemius muscle [28].
- Cost-effectiveness analysis: A cost-effectiveness analysis will be performed comparing the two treatment protocols.
- Re-rupture.
- Perimeter of calf [29].
- Type of work and return to work.
- Type of sport and return to sport.
- Achilles tendon resting angle (ATRA) [29].
- Achilles tendon length measure (ATLM).
- Plantar flexion power is measured with a hand-held dynamometer fixed to the examination bed with a strong Velcro band.
- Deep vein thrombosis (DVT) screening by Doppler ultrasonography.

### Study participants

Patients who are treated for acute Achilles tendon rupture at Copenhagen University Hospital Hvidovre are eligible for inclusion if they fulfill the inclusion criteria. Patients who do not wish to participate are treated non-operatively without early controlled motion of the ankle joint.

#### Inclusion criteria

The inclusion criteria are as follows:

- Age 18–70 years.
- The patient must be expected to be able to attend rehabilitation and post-examinations.
- The patient must be able to speak and understand Danish.
- The patient must be able to give informed consent.

#### Exclusion criteria

The exclusion criteria are as follows:

- Former rupture of one or both Achilles tendon(s).
- Previous surgery on the Achilles tendon.
- Fluoroquinolone treatment within the last 6 months.
- Tendinosis treated with corticosteroids (tablets or injections) within the last 6 months.
- Diagnosis of arterial insufficiency in the legs.
- Terminal illness or severe medical illness: American Society of Anesthesiologists (ASA) score higher than or equal to 3.
- The space between the rupture and the calcaneus is less than 1 cm.

### Recruitment organization

The day after primary treatment at the emergency room, patients are attended to in the outpatient clinic. The patients are examined by an investigator, who assesses whether the patient meets the inclusion criteria. If so, the patient is verbally informed of the study and is given written patient information. The patient is given the opportunity — on an informed basis without any pressure — to decide whether he/she wants to participate in the trial. The patient is informed of his/her right to 24 hours of reflection.

### Randomization

Participants will be randomly assigned to either the control or experimental group with a 1:1 allocation as per a computer-generated randomization. An experienced senior researcher with no other connection to the trial is responsible for generation of the allocation key and creation of numbered, opaque, and sealed envelopes. The allocation key is stored by and only accessible to the senior researcher. If a participant needs to know the allocated treatment ahead of trial termination, this will be arranged by the project nurse. Randomization is performed by the project nurse.

### Blinding

The physiotherapists who conduct the follow-up examinations and the primary investigator conducting the data analysis are blinded to the intervention. Patients and investigators are not blinded to delivery of the intervention and control. All patient contact in the treatment period from weeks 2 to 8 is coordinated by a project nurse, a physiotherapist, and an orthopedic surgeon with no connection to the follow-up visits and data analysis.

### Time schedule

Recruitment of patients for the study began in February 2014 and was finished in November 2016. Final follow-up will be conducted in November 2018.

### Setup

#### Place of investigation

The trial is taking place at Copenhagen University Hospital Hvidovre, Denmark. At this hospital patients with acute Achilles tendon rupture are recommended non-operative treatment with the following exceptions: (1) ruptures older than 5 days, (2) degenerative ruptures (patients treated with steroids or fluoroquinolones within the last 6 months), and ruptures within 1 cm of calcaneus.

#### Intervention

**Fig. 1:**
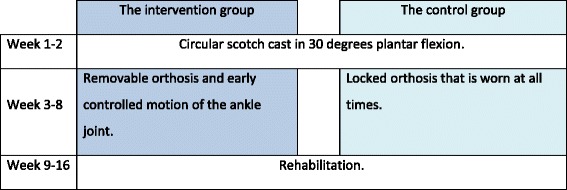
Study design

**Fig. 2:**
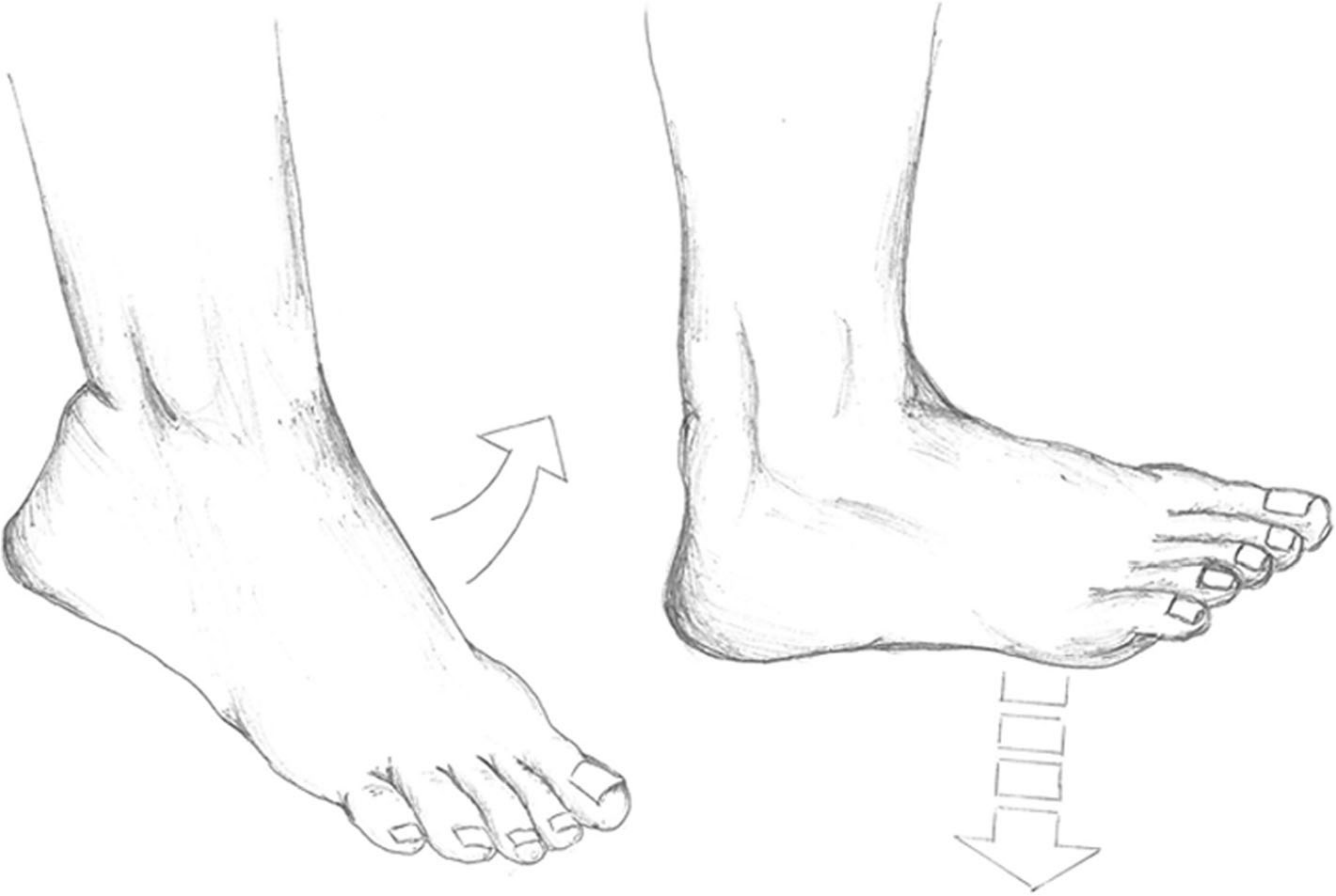
The intervention: early controlled motion of the ankle joint. The orthosis is removed with the patient sitting on the edge of a table with both legs hanging. Gravity bends the foot downward, whereupon the patient must actively flex the foot upwards to a horizontal position. This is done at least five times a day in series of 25 repetitions

A cast is applied in the emergency room and changed to a circular scotch cast in the outpatient clinic within 4 days. No weight bearing on the injured leg is allowed within the first 2 weeks post-injury. After 2 weeks the cast is changed to a removable orthosis, and full weight bearing is allowed. At this point patients who choose to participate in the trial are, through randomization, placed in one of the following two groups (see Fig. 1): (1) the intervention group, which must perform controlled ankle motion exercises from the beginning of week 3 through week 8 or (2) the control group; in line with the current treatment regimen, the patients must keep the orthosis on at all times, and they are not allowed to move the ankle before week 9. The treatment protocols for the two groups are similar except for the intervention: early controlled motion of the ankle (Fig. 2).

#### Compliance

To ensure compliance within the groups, the patients of the intervention group are equipped with training diaries in which they record their training for each of the five daily training sessions. The patients in the control group have their orthosis sealed with a plastic strap designed for the purpose. If the orthosis is removed between clinical follow-ups, it is registered by the project nurse.

### Detailed description of the examination and treatment weeks 0–8

The following treatment protocol is similar for both groups.

Day 0: In the emergency department the diagnosis is made based on the following criteria: (1) a patient history with a clear sense of ”snap” of the Achilles tendon, (2) a palpable defect, usually located 3–6 cm above the calcaneal tubercle, and (3) a positive calf squeeze test [30]. A plaster of Paris is applied to the patient’s foot in a plantigrade position (30–45 degrees). No weight bearing is allowed.

Days 1–3: The diagnosis is confirmed by a physiotherapist specialized in treatment of acute Achilles tendon rupture, and the foot is put in a circular scotch cast with the foot in a plantigrade position. Verbal and written information on the project is delivered to the patient.

Day 14: The cast is changed to a removable orthosis (AirCast AirSelect Standard from DJO Global, Vista, CA, USA) with two 1.5-cm heel lift wedges. Full weight bearing is allowed, but the patient is advised to use crutches for another week or two. Randomization is performed.

Week 4: The patient is seen by the project nurse, and the first wedge is removed. For the intervention group, the training diary is inspected and patients are encouraged to do their home exercises. For the control group, the seal is broken and the foot is gently washed before reapplying the seal.

Week 6: The patient is seen by the project nurse, and the second and last wedge is removed. For the intervention group, the training diary is inspected and patients are encouraged to do their home exercises. For the control group, the seal is broken and the foot is gently washed before reapplying the seal.

Week 8: The orthosis is removed, and the tendon is inspected. Tendon healing is examined clinically and by ultrasound. During the following 4 weeks, crutches are used as needed, and the orthosis is used in situations of high strain/risk.

### Detailed description of the rehabilitation weeks 9–16

Rehabilitation from week 9 to 16 is the same for the two groups, and is organized as group exercises twice a week at the hospital. A standardized rehabilitation program inspired by Willits et al. and Nilsson-Helander et al. is used (Table 1) but adjusted individually if needed [19, 20]. The sessions are led by physiotherapists and last for 60 minutes.

**Table 1:** Rehabilitation weeks 9–16

Basic exercises
Exercise bike: 10–15 min
Ankle range of motion (pronation and supination, dorsal and plantar flexion. Dorsal flexion to 90 degrees. The other movements unlimited): 2 × 8 reps
Standing heel-rise (2 × 3 s tempo) 3 × 10 reps^a^
One leg standing balance exercise: 3 × 30 s^a^
Weeks 9–11
Basic exercises
Exercises with resistance band around the foot in sitting position (knee extended, dorsal flexion to 90 degrees, plantar flexion and inversion): 2 × 20 reps
Side laying hip abduction: 2 × 15 reps
Heel-rise in supine position with flexed legs: 2 × 15 reps
Sitting heel-rise with weight on injured leg (20–25 repetitions maximum): 3 × 15 reps
Gait training
Once a week, the training is situated in a pool where similar exercises are performed.
Weeks 12–16
Basic exercises (as above)
Walk on toes with support to start with, if needed: 2 × 5 m
Standing heel-rise is performed with increased weight on injured leg: 5 × 10 reps
Heel-rise in supine position with flexed legs (with increased weight on injured leg): 2 × 15 reps
Leg press with one leg at a time (10 repetitions maximum): 2 × 10 reps
Balance exercise on a trampoline: 2 × 45 s
Walk/jog on a trampoline: 2 × 45 s
Cross trainer: 1 min and 45 s
Lunges (only with injured leg in front): 2 × 10 reps
The plank (core exercise): 2 × 45 s
This program is performed as circle training.
Return to running—from week 14
Jogging upwards on stairs is allowed when the patient can walk 5 m on toes without heel falling down.
Running on even ground is allowed when the patient can perform 5 single-legged heel-rises with approximately 90% of the height of the un-injured leg.
After completed rehabilitation program
Examination of tendon healing and function. If needed, referral to further physiotherapy. Gradual return to sports (contact sports earliest 6–9 months after injury)

^**a**^Home exercises, three times daily

### Follow-up investigations

**Fig. 3:**
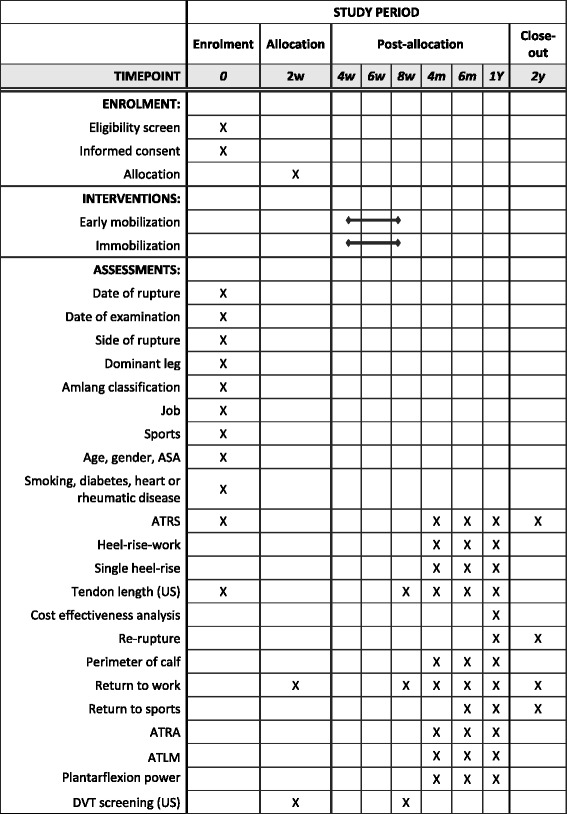
SPIRIT table of enrollment, interventions, and assessments

Follow-up is performed at 4 months, 6 months, 1 year, and 2 years (Fig. 3). The study’s primary endpoint is at the 12 months follow-up. Follow-up is performed by blinded physiotherapists. Before entering the examination room, the patients are reminded by a secretary not to reveal which treatment group they belong to.

Patients who discontinue the treatment are still encouraged to participate in the follow-up. They are contacted up to three times via mail or telephone.

### Registration and availability of data

All relevant data are recorded in specially designed case report files. The patients are identified by an assigned number. At the completion of the study, all identifiable data will be destroyed. The patients are informed, both verbally and in writing, that data are stored and analysed in a computer, that the patient’s anonymity is preserved, and that the data protection legislation is adhered to. The data registration is reported to the Danish Data Protection Agency, identifier HVH-2014-002, I-Suite no. 02608. The list of registered data is included in Fig. 3. There will be free access to the final anonymized trial dataset. Due to the limited size of the study and the safety of the treatments, a data monitoring committee is not considered necessary.

### Statistics and analysis

#### Sample size

Sixty-five patients will be included in each group, thus 130 patients in total. The sample size calculation is based on a clinical relevant difference of 10 points in ATRS, a standard deviation (SD) of 16, and power of 0.90 (two sided). In a previous study with a fully comparable population, we found an SD of 16 points at the 1 year post-examination [1]. Fifty-four patients are required in each group; due to the risk of dropout, 65 patients will be included in each group.

#### Analysis of endpoints

The two groups are described with regard to demographic parameters as well as primary and secondary endpoints. The primary and secondary outcomes of both groups are compared by the use of relevant statistics according to the characteristics and distribution of the variables. The change of the outcomes over a period of time is described within each group.

All statistical testing will be performed at the two-sided 5% significance level, and 95% confidence intervals will be presented where appropriate. No formal interim analyses are planned, and hence no statistical testing will take place until the 1 year analysis. The 1 year analysis will take place after all participants have completed their 1 year follow-up and sufficient time has been allowed for data entry and validation.

Prior to any analysis, missing data patterns will be investigated and reasons for missing data obtained and summarized where possible. The primary analysis will be conducted as an intention-to-treat analysis, which includes all participants with missing outcome data, unless there is clear evidence that its underlying assumption is inappropriate. A sensitivity analysis will be performed to assess the robustness of the results by imputing missing data using multiple imputation under both missing at random and missing not at random assumptions.

### Safety

#### Risks and side effects

Non-operative treatment of Achilles tendon rupture with or without early controlled motion of the ankle joint is a safe treatment used in orthopedic departments all over the country. At Copenhagen University Hospital Hvidovre we have periodically used first one and then the other regimen without registered risks and side effects for patients. One can theoretically argue that patients allowed early movement of the ankle joint are at risk of healing with an elongation of the Achilles tendon. Moreover, there is always the risk of unknown side effects. Patients participating in the trial are covered by the patient insurance of Copenhagen University Hospital Hvidovre.

#### Adverse events

In this context an adverse event is defined as any unintended, unfavorable finding, symptom, or disease that occurs, whether it is considered to be related to the study or not. Adverse events will be recorded.

#### Critical adverse events

In this context a critical adverse event is defined as an event or reaction which will cause: death, life-threatening situations, hospitalization or prolongation of existing hospitalization, or permanent or severe disability.

Critical adverse events must be assessed by an investigator regarding possible correlations with the trial’s modified treatment in order to consider whether there is a reasonable possibility that this caused the adverse event. The following factors are included in the assessment: consistency in time, consistency with the known effects of treatment, and alternative causes.

If a critical adverse event is considered to have a causal relationship with the treatment, then the project manager and other clinically responsible investigators will evaluate whether the study should be terminated.

### Satellite studies

Separate protocols have been written for the following substudies.

#### Ultrasonographic-guided treatment of acute Achilles tendon rupture: evaluation of two novel ultrasonographic measurements

This study will validate Amlang’s ultrasound classification and investigate whether the treatment of acute Achilles tendon ruptures can be guided by (1) Amlang’s ultrasound classification of acute Achilles tendon rupture [31] or (2) Barfod’s ultrasound measurement to determine the elongation of the Achilles tendon following a rupture [28]. The study is registered at ClinicalTrials.gov: NCT02062567.

#### Metabolic complications following Achilles tendon rupture

This study will evaluate the effect on glucose, lipid, and bone metabolism following conservative orthopedic procedures in patients who suffered from acute Achilles tendon rupture. We want to investigate the putative negative impact on glucose, lipid, and bone metabolism during a period of restraint from exercise secondary to immobilization following Achilles tendon rupture. We also want to study these patients during their physical active rehabilitation (at weeks 8–52) to establish whether they succeed in improving the metabolic impairments they suffered during the early post-injury period, during which strong limitations on physical activity are prescribed (weeks 0–8). The study is registered at ClinicalTrials.gov: NCT02015364.

#### Risk of deep vein thrombosis in non-operative treatment for acute Achilles tendon rupture

This study will investigate the risk of deep vein thrombosis (DVT) in non-operative treatment for acute Achilles tendon rupture in the presence of a risk-stratified protocol for antithrombotic treatment. All study patients are investigated for DVT by use of blood samples and Doppler ultrasonography performed by an experienced radiologist at 2 and 8 weeks.

## Discussion

### The intervention

The content of the intervention was carefully selected based on the experience of a finished Ph.D. study [32]. Early controlled motion of the ankle can be performed in different ways: some use hinged orthoses allowing for movement of the foot while walking and resting [11, 19], while others use fixed orthoses that are to be removed for allowance of movement of the ankle [1]. In the time period leading up to this study, different orthoses were tried and evaluated in the department. A fixed orthosis was chosen, as it seemed the most reliable choice in the study setup. The chosen motion protocol is based on the one developed by Willits et al. [20]. It is, however, debatable how often the ankle is to be taken out of the orthosis and how many exercises should be performed. The hinged orthoses were opted out due to technical problems with adjustment of the center of rotation and anxiety of putting too large a strain on the healing tendon.

### Endpoints

For this study the patient-reported outcome measure ATRS [26] was chosen as the primary endpoint. It is validated in a Danish context [33] and has been increasingly used since its publication in 2007 [1, 19, 34]. The score reports the patient perception of the function of the healed tendon and not the actual function of the tendon. The incidence of Achilles tendon ruptures peaks in the early 40s [3], the age period in which many people reduce their physical activities and change their pattern of exercise and sporting activities. Therefore, patients might not register impairments leading to decreased physical capabilities, and a high ATRS might be due to low demands of the tendon and not a well-functioning tendon. Following this argument, a physical endurance test like the heel-rise work test [35] or a measurement of tendon length [28, 29] might constitute better primary endpoints. However, these measurements are very specific and do not describe the tendon’s actual capability to function in everyday life. Thus, the ATRS was chosen for this study.

### Compliance

Compliance to the allocated treatment protocol is important in order to be able to measure the effect of the intervention. In the present study, compliance is measured with a training diary in the intervention group and a seal of the orthosis in the control group. The possibility of measuring compliance with use of motion sensors was investigated prior to the trial but found impossible.

### Changes to the protocol

Minor changes were performed between the first and the second versions of the protocol. The first version, dated 29 November 2013, was used for institutional review board (IRB) approval and clinical trial registration. The changes did not affect the essential parts of the protocol and did not lead to any changes in the trial registration. The second version of the protocol was accepted by the Ethical Review Board of the Capital Region of Denmark on 5 August 2014.

### Trial status

The trial commenced in February 2014. Recruitment was originally expected to span 2 years, but due to initial slow recruitment it is now expected to span 2½ years. By the end of February 2016, 110 of 130 patients had been included.

## Additional file

Additional file 1: SPIRIT 2013 Checklist: Recommended items to address in a clinical trial protocol and related documents*. (DOC 122 kb)

## Abbreviations

ASA: American Society of Anesthesiologists
ATLM: Achilles tendon length measure
ATRA: Achilles tendon resting angle
ATRS: Achilles tendon Total Rupture Score
CONSORT: Consolidated Standards of Reporting Trials
DVT: Deep vein thrombosis
RCT: Randomized controlled trial
SD: Standard deviation
SPIRIT: Standard Protocol Items: Recommendations for Interventional Trials
